# Retrospective Cohort Study: Severe COVID-19 Leads to Permanent Blunted Heart Rate Turbulence

**DOI:** 10.3390/diagnostics15050621

**Published:** 2025-03-05

**Authors:** Mücahid Yılmaz, Çetin Mirzaoğlu

**Affiliations:** Department of Cardiology, Elazığ Fethi Sekin City Hospital, University of Health Sciences, 23280 Elazığ, Turkey; dr.cmirzaoglu@gmail.com

**Keywords:** recovery from severe COVID-19, blunted heart rate turbulence, autonomic nervous system disorder, ventricular arrhythmia

## Abstract

**Background:** Heart rate turbulence (HRT) is a non-invasive technique that can be used to evaluate autonomic nervous system (ANS) function and cardiac arrhythmia. The objective of this study is to investigate whether COVID-19 can lead to long-term blunted HRT following recovery. **Methods:** This retrospective cohort study included 253 individuals with a confirmed history of COVID-19, referred to as the recovered COVID-19 group, along with 315 healthy participants who had no history of the virus. The recovered COVID-19 group was categorized into three subgroups based on their chest CT severity scores. The HRT analyses were obtained from a 24-h electrocardiography-Holter recording. **Results:** This study revealed that the HRT onset value was elevated in the recovered COVID-19 group, while the HRT slope value showed a significant decrease when compared to the control group. Correlation analyses indicated a positive relationship between the chest CT severity score and HRT onset, whereas a negative correlation was observed between the chest CT severity score and HRT slope. Regression analyses identified recovery from severe COVID-19, chest CT severity score, hypertension (HT), and smoking as independent predictors of both abnormal HRT onset and the existence of an abnormal HRT slope. **Conclusions:** Individuals who have recovered from severe COVID-19 are expected to encounter a permanent blunting of HRT, which is regarded as a significant indicator of an increased risk of ventricular arrhythmias and impaired autonomic nervous system (ANS) function. Recovered severe COVID-19 individuals should be carefully evaluated for HRT with 24-h ECG-Holter.

## 1. Introduction

SARS-CoV-2 (COVID-19), first revealed in the People’s Republic of China in December 2019, has spread globally, resulting in severe levels of mortality and morbidity. The disease has a clinical spectrum ranging from a mild form, in which it can progress asymptomatically, to multi-organ failure, which results in death. Although it mainly affects the respiratory tract, other systems, including the neurological and cardiovascular systems, are also affected by this infection [1].

Heart rate turbulence (HRT) originates from the principle of accelerating sinus rhythm, which occurs as a reflex against hypotension, which occurs as a result of the inability of the entire ventricle to fill with blood due to the short diastole period during ventricular premature beats in patients with sinus rhythm. In a healthy heart, this brief hypotension after a premature beat triggers an increase in heart rate, with a defined degree of acceleration. HRT indicates this degree of acceleration. In other words, it shows the autonomic activity of the heart. For a healthy HRT, both the sympathetic and parasympathetic pathways of the heart must be intact [2]. Abnormal heart rate turbulence (HRT) characterizes patients with autonomic dysfunction or reduced baroreflex sensitivity resulting from various conditions. It is well established that diminished HRT serves as an indicator for patients at elevated risk of both sudden and all-cause mortality [3,4].

Recent observations indicate a rise in hospital admissions among patients post-COVID-19 recovery, presenting with symptoms of heart palpitations. These patients are frequently redirected to another department with an initial diagnosis of somatization or panic disorder. Could a more sensitive assessment be made for these individuals? Is there proof that SARS-CoV-2 leads to lasting damage to the autonomic nervous system (ANS)? The prolonged consequences of SARS-CoV-2 on cardiac autonomic function in people who have successfully recovered from COVID-19 were investigated in this research using heart rate turbulence (HRT).

## 2. Materials and Methods

### 2.1. Study Population

**Figure 1:**
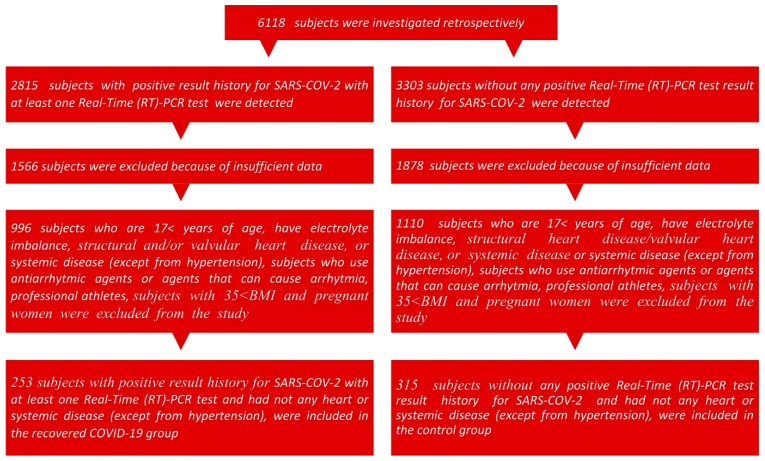
The subject inclusion flowchart diagram.

A total of 6118 patient files of those who visited the cardiology outpatient clinics of Elazığ Fethi Sekin City Hospital from 1 January 2021 to 1 May 2023 and underwent 24-h electrocardiographic (ECG)-Holter monitoring were retrospectively analyzed. Patients with incomplete information in their records and those who are under the age of 17, individuals with structural heart disease or systemic diseases (excluding hypertension), as well as patients taking anti-arrhythmic medications or drugs that may induce arrhythmias, professional athletes, individuals with a body mass index (BMI) greater than 35, and women who were pregnant were excluded from the research. This study comprised a total of 253 individuals with a documented history of positive SARS-CoV-2, as confirmed by at least one real-time (RT)-PCR test conducted on nasopharyngeal swabs using the Bio-Rad CFX96 real-time PCR Detection System (Bio-Rad Laboratories, Inc., Hercules, CA, USA). These individuals were classified as having recovered from COVID-19. In addition, the control group consisted of 315 participants who did not show any positive RT-PCR test results for SARS-CoV-2 throughout the same interval. Furthermore, evaluations, including laboratory tests, patient histories, and physical examinations, indicated that these subjects had no cardiac or systemic diseases (with hypertension excluded from this assessment) (Figure 1).

Individuals in the cohort who successfully recovered from COVID-19 had experienced SARS-CoV-2 infection at least once, with a maximum of three infections. The interval between the positive result of the last RT-PCR test and the 24-h ECG-Holter assessment for subjects in the recovered COVID-19 group varied, ranging from as little as 4 weeks to as long as 144 weeks.

Among those who had recovered from the virus, a chest computed tomography (CT) severity score was applied. The extent of involvement in each of the five lung lobes (three lobes on the right and two on the left) was assessed visually. The score corresponding to the involvement percentage of each lobe was multiplied by the number of affected lobes, resulting in a Chest CT severity score ranging from 0 to 25 for each participant (Table 1) [5].

The cohort of individuals who had recovered from COVID-19 was divided into three subgroups according to their chest CT severity scores, which are defined as follows:

➢ Subgroup I (Mild) (155 subjects): This subgroup consisted of individuals with a chest CT severity score of less than 8.
➢ Subgroup II (Moderate) (56 subjects): Individuals with chest CT severity scores ranging from 8 to 15 were categorized in the moderate subgroup.
➢ Subgroup III (Severe) (42 subjects): Those with a chest CT severity score between 16 and 25 were classified in the severe subgroup.

The current investigation adhered to the ethical standards set forth in the Declaration of Helsinki. This study received approval from the Ethics Committee of T.C. Fırat University (Approval No.: 2021/10-38). Given the retrospective nature of the research, the requirement for patient consent was waived.

### 2.2. 24-Hour ECG-Holter Monitoring

For the analysis of heart rate turbulence (HRT), 24-h ECG-Holter recordings were analyzed utilizing a 4-lead Holter device (Digitrak XT, Philips Medical Systems, Andover, MA, USA) alongside Cardioscan II premier software, firmware version C.2. The HRT parameters were determined following the methodology established by Bauer et al. [6]. Two important numerical metrics were utilized for measurement: turbulence onset (TO), representing the beginning of the acceleration phase of sinus rhythm, and turbulence slope (TS), which reflects the deceleration phase. Heart rate turbulence (HRT) was assessed by measuring the percentage variation between the average duration of the first two RR intervals that occurred after the compensatory pause following a premature ventricular complex (PVC) and the average duration of the last two RR intervals that were recorded just before the PVC event. This was calculated using the formula: TO = [(RR1 + RR2) − (RR-2 + RR-1)/(RR-2 + RR-1)] × 100 (%). Here, RR-2 and RR-1 represent the two RR intervals immediately before the PVC, while RR1 and RR2 refer to the two RR intervals directly after the compensatory pause. The turbulence slope was defined as the maximum positive slope of a regression line calculated across any five consecutive sequences within the first 15 sinus intervals following a PVC, expressed in milliseconds per beat. An HRT onset of ≥0% and an HRT slope of ≤2.5 ms/beat were categorized as abnormal [7].

The subjects were classified into three distinct categories: category 0 for those with normal values for both HRT onset and HRT slope; category 1 for individuals exhibiting an abnormality in either one of the two parameters; and category 2 for those with abnormalities in both metrics. In cases where HRT cannot be determined due to the absence of, or insufficient, suitable ventricular premature complex tachograms in the recordings, individuals in sinus rhythm were assigned to HRT category 0 [7].

### 2.3. Statistical Evaluation

Statistical analysis utilized SPSS software, version 27.0, from SPSS Inc., located in Chicago, IL, USA. The Kolmogorov–Smirnov test was employed to evaluate the normal distribution of continuous variables. Given that all continuous variables showed a non-normal distribution, Tamhane’s T2 correction was applied within the one-way ANOVA test to analyze these parameters, reporting results as medians with the 25th and 75th percentiles. Baseline characteristics among the groups were compared through the chi-square test for categorical variables, which were reported as frequencies and percentages. To analyze correlations between continuous variables, both Spearman’s rho and Pearson’s correlation analyses were utilized. Furthermore, binary logistic regression and linear regression analyses were performed to identify clinical variables that could independently predict the presence of abnormal HRT onset and slope. A *p*-value below 0.05 was considered to be statistically significant.

## 3. Results

**Figure 2:**
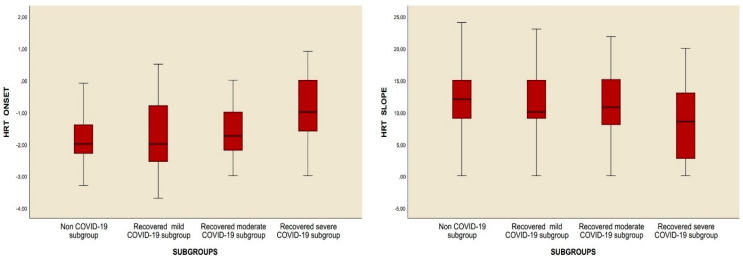
Comparison of HRT onset and HRT slope values between the subgroups.

This study included 253 patients who had overcome the infection and 315 healthy subjects without any known systemic disorders, excluding hypertension. HRT slope and percentage of category 0 were found to be significantly decreased; on the other hand, HRT onset was increased in the recovered COVID-19 group when compared to the control group (Table 2). There was no statistically significant difference in age, hypertension (HT), gender, smoking, percentages of abnormal HRT onset, abnormal HRT slope, category 1, and category 2 between the recovered COVID-19 group and the controls (Table 2). However, when subgroup analysis was performed, it was observed that the HRT slope value was decreased and the HRT onset value was increased. In addition to the percentages of abnormal HRT onset and abnormal HRT slope, the percentages of category 1 and category 2 were significantly higher in subgroup 3 compared to the control group. Moreover, while no difference was observed in any HRT parameters reported above between the control group and subgroups 1 and 2, the percentages of abnormal HRT onset, abnormal HRT slope, percentages of category 1 and category 2, and HRT onset value were significantly higher, and the HRT slope value was significantly decreased in subgroup 3 compared to subgroup 1. Last, compared to subgroup 2, the HRT onset value and abnormal HRT onset percentage increased in subgroup 3, while the HRT slope and category 0 percentage decreased. No difference could be detected between subgroup 2 and subgroup 3 as regards the percentage of category 1 and category 2 (Table 3, Figure 2).

**Figure 3:**
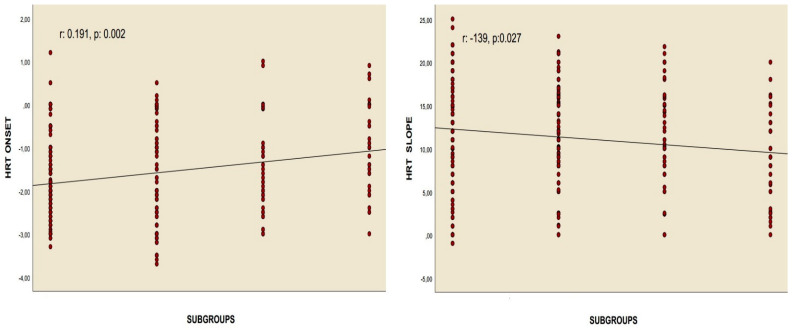
Spearman’s rho correlation analyses between HRT onset, HRT slope, and subgroups.

**Figure 4:**
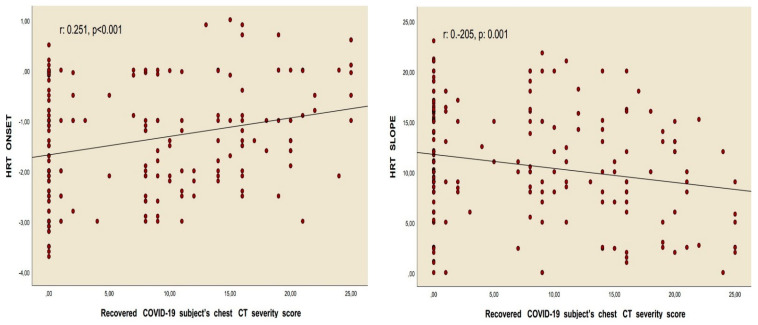
Pearson’s correlation analyses between HRT onset, HRT slope, and recovered from COVID-19 subject’s chest CT severity score.

Correlation analyses revealed a positive relationship between the chest CT severity score and HRT onset values in patients who had overcome the infection, as well as a negative association between the chest CT severity score and HRT slope values, assessed using Pearson’s correlation. Similarly, Spearman’s rho correlation test identified a positive correlation between the recovered COVID-19 subgroups and HRT onset, alongside a negative correlation with the HRT slope. Nonetheless, no meaningful correlations were identified between these values and the quantity of positive PCR tests for COVID-19, nor with the length of time since the infection occurred (Table 4, Figure 3 and Figure 4).

The findings from both binary and linear regression analyses revealed that certain factors served as independent predictors of abnormal HRT onset and abnormal HRT slope. These factors included recovery from severe COVID-19, the chest CT severity score in individuals who had recovered, as well as the presence of hypertension and smoking status (Table 5, Table 6, Table 7 and Table 8).

Upon examining Model 1, it was found that the logistic model designed to assess the impact of independent variables on the prediction of abnormal HRT onset was statistically significant (Nagelkerke R^2^ = 0.252; *p* < 0.001). Based on this model, recovery from severe COVID-19 was recognized as a risk factor for abnormal HRT onset, with a likelihood of 5.435 times greater than that of other factors when compared to the control group and other subgroups (Table 5). In the evaluation of Model 2, the established logistic regression model also demonstrated statistical significance (Nagelkerke R^2^ = 0.234; *p* < 0.001). Within this model, recovery from severe COVID-19 was found to be a 4.756 times risk factor for the presence of an abnormal HRT slope in comparison to the control group and other subgroups (Table 6).

Analysis of Model 3 revealed that the developed linear regression model demonstrated statistical significance (Nagelkerke R^2^ = 0.345; *p* < 0.001). This model indicated that for every 1-point increase in the chest CT severity score, the HRT onset value increased by 0.035 (Table 7). In the case of Model 4, it was similarly found that the established linear regression model exhibited significance (Nagelkerke R^2^ = 0.318; *p* < 0.001). According to this model, a 1-point increase in the chest CT severity score was associated with a decrease of 0.122 in the HRT slope value (Table 8).

## 4. Discussion

The results of this study showed that the HRT slope value was notably reduced in subgroup 3 when compared to the control and other groups. In contrast, the HRT onset value, the percentage of abnormal HRT onset, the percentage of abnormal HRT slope, and the proportions of categories 1 and 2 were significantly elevated (Table 3, Figure 2). Correlation analyses demonstrated a significant relationship between the chest CT severity score and both the HRT onset and HRT slope values. However, no relationship was identified between these parameters and either the number of recovered COVID-19 cases or the duration since recovery (Table 4, Figure 3 and Figure 4). Additionally, regression analyses identified recovery from severe COVID-19, the chest CT severity score of recovered COVID-19 patients, hypertension, and smoking status as independent predictors of both HRT onset and HRT slope (Table 5, Table 6, Table 7 and Table 8).

**Figure 5:**
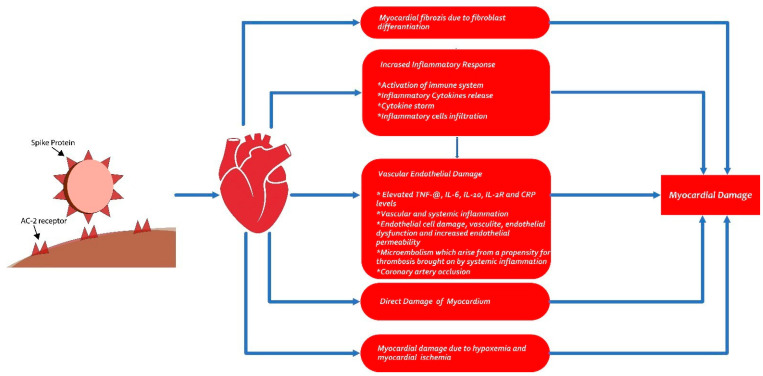
Schematic presentation of myocardial damage in COVID-19.

The infection caused by SARS-CoV-2 occurs when the spike proteins on the surface of the virus interact with the angiotensin-converting enzyme 2 (ACE-2) receptor. The widespread presence of the ACE-2 protein across various tissues—including the myocardium, bone marrow, central nervous system, kidneys, gastrointestinal tract, epithelial cells, and spleen, particularly in type 2 pneumocytes—provides insight into the multi-organ damage often linked to the infection of SARS-CoV-2 [8]. It is hypothesized that myocardial damage during COVID-19 may occur through two mechanisms: (a) Cytokine storm manifested by increased ferritin, lactate dehydrogenase (LDH), interleukin (IL)-6, and D-dimer, accompanied by increased high-sensitivity troponin I values. An unbalanced response in T helper cells, hypoxia-induced excessive increase in intracellular calcium in cardiac myocytes, and development of apoptosis are observed in this situation. (b) The effect of the SARS-CoV-2 virus directly on the myocardium via the ACE-2 pathway. The existence of ACE-2 receptors in myocardial tissue and vascular endothelial cells suggests that myocarditis may occur by direct virus infection of myocardial tissue, theoretically [9,10]. Another possible mechanism, as shown in the pathological examinations performed in those suffering from severe acute respiratory syndrome coronavirus (SARS-CoV-1), is that vasculitis may occur with monoxide and lymphocyte infiltration into arterial and venous endothelial cells where ACE-2 receptors are intense. The resulting vascular endothelial damage may cause stromal edema in the heart [11]. The virus entering the myocardial endothelium can trigger vasculitis directly, and the presence of the virus in the body can cause a hypersensitivity reaction with an indirect immunological response (Figure 5) [12,13].

Although SARS-CoV-2, the last identified member of the coronavirus family, primarily affects the respiratory tract, experimental studies and case reports bring up the neurotropic effect of the virus [14]. There are two primary explanations for how SARS-CoV-2 gains access to the central nervous system (CNS): (a) The virus may move from systemic circulation to cerebral circulation by damaging the capillary endothelium, facilitated by reduced blood flow. (b) Alternatively, it is believed that the virus can directly enter the CNS through the cribriform plate and olfactory bulb, potentially affecting the cerebrospinal fluid (CSF) and, by extension, the vagal nerve [15,16]. ACE-2 receptors, which are an important target for SARS-CoV-2, are found in the CNS as well as in many organs [8]. Therefore, it seems possible for SARS-CoV-2 to have CNS effects. In addition, high expression of ACE-2 receptors in endothelial cells may cause cerebral edema and intracranial hypertension findings by impairing blood–brain barrier permeability [17]. Research has demonstrated that the virus can penetrate the central nervous system (CNS) through the olfactory nerve, presenting an alternative route for infection. Once in the CNS, the virus can propagate from one neuron to another via axonal transport mechanisms [18]. In addition to this direct invasion of the nervous system, COVID-19 can lead to neurological complications that arise not only from the virus itself but also as a consequence of extensive cardiopulmonary failure and metabolic disturbances caused by SARS-CoV-2 infection. Furthermore, these neurological issues may also be linked to autoimmune responses triggered by the infection, highlighting the multifaceted ways in which COVID-19 can impact neurological health [19]. In particular, the cytokine storm that occurs during the disease activates T lymphocytes, macrophages, and endothelial cells by increasing inflammatory cytokines. Then, increased release of interleukin (IL)-6 causes neuron/brain damage by activating vascular leakage, complement activity, and the coagulation cascade [14].

Although many drugs are used in the treatment of COVID-19, corticosteroids (prednizolon, cortisone, dexamethasone, etc.) attract attention with their potential to cause serious damage to the myocardium due to long-term use. Corticosteroids can be given to patients with severe COVID-19 in significantly elevated doses over an extended period to prevent the development of acute respiratory distress [20]. Hypercortisolism contributes to an increase in myocardial mass through elevated microvascular density and enhanced total water and fat content within the myocardial structure [21]. Beyond their glucocorticoid effects, corticosteroids exhibit some mineralocorticoid characteristics as well. Mineralocorticoids promote collagen secretion by activating fibroblasts, which can lead to the development of diffuse myocardial fibrosis in individuals with hypercortisolism [21,22]. The mineralocorticoid effect also causes the migration of macrophages into the perivascular area and extracellular matrix. Additionally, systemic hypervolemia caused by the renal effects of mineralocorticoid receptors further increases myocardial edema [23,24].

Heart rate turbulence (HRT) is a phenomenon observed in electrocardiography that reflects temporary hemodynamic disturbances caused by ventricular premature beats, characterizing baroreflex-mediated short-term variations in sinus cycle length following these spontaneous beats [6]. In individuals with normal physiology, the sinus rate experiences a brief acceleration before returning to its baseline level, which is lower than the rate prior to the ventricular premature beats, followed by a subsequent deceleration. The transient decrease in blood pressure due to the premature beat activates baroreceptors, leading to an increased heart rate through vagal inhibition and a reduction in the lengths of RR intervals as indicated by turbulence onset (TO). Concurrently, the relative hypotension triggers sympathetic activation within the autonomic nervous system [25]. This heightened sympathetic response leads to an incremental increase in systolic blood pressure and vascular resistance. Consequently, vagal activity increases again, causing a prolongation of cycle lengths, which is assessed by turbulence slope (TS) [6,25,26]. Thus, heart rate turbulence necessitates a well-coordinated interaction between the vagal and sympathetic nervous systems. Any alteration in either of these systems can lead to abnormalities in heart rate turbulence [26].

Sudden cardiac death, mainly resulting from ventricular tachyarrhythmias, continues to be the leading factor contributing to mortality. However, identifying the factors that lead to ventricular fibrillation and developing safe and effective antiarrhythmic medications remain a challenge. A combination of diminished parasympathetic regulation and heightened sympathetic activation may create conditions favorable for malignant ventricular arrhythmias [27]. Both the turbulence slope (TS) and turbulence onset (TO) parameters are linked to baroreflex sensitivity and the balance of the autonomic nervous system [28,29]. Bauer et al. introduced a novel non-invasive risk factor for sudden death in electrocardiology known as heart rate turbulence. This physiological occurrence is characterized by an initial increase in sinus rhythm that is subsequently followed by a decrease after a premature ventricular complex. Heart rate turbulence is believed to reflect the sensitivity of the baroreflex. Both retrospective and prospective studies have shown that heart rate turbulence (HRT) acts as a crucial and independent indicator of mortality following a myocardial infarction [6,28,29,30]. If changes in cardiac autonomic regulation are crucial in the genesis of life-threatening arrhythmias, it can be anticipated that interventions aimed at enhancing parasympathetic activity or reducing cardiac adrenergic activity may also offer protection against ventricular fibrillation [27].

Long COVID-19 is characterized by complaints such as palpitations, persistent fatigue, alterations or loss of smell, chest and muscle pain, and dyspnea that cannot be attributed to any other disease, persisting for at least 12 weeks following acute COVID-19 infection [31,32,33]. Some surveys have indicated that patients recovering from COVID-19 report a higher frequency of palpitations and tachycardia compared to individuals who have never contracted the virus [31,34].

However, the way patients describe their palpitations can differ based on the questionnaire’s design, and the results typically reflect a subjective interpretation of the participants’ experiences. A recent study conducted via an online questionnaire proposed that postural orthostatic tachycardia syndrome may be the underlying cause of the complaints reported by the respondents [34]. Nonetheless, there remains a clear need for studies grounded in objective electrocardiographic data to demonstrate that these subjective complaints are indicative of specific cardiac arrhythmias.

In this study, the values for HRT slope and HRT onset, as well as the number of patients exhibiting abnormal HRT, showed marked differences in subgroup 3 compared to controls and other subgroups (Table 3). Abnormal HRT values were found to be associated with subgroup 3 and the chest CT severity score of the participants, but not with the number of positive PCR tests or the amount of time that had passed since recovery, according to correlation and regression analyses (Table 4). Ultimately, the findings revealed that both subgroup 3 and the chest CT severity score were independent predictors of abnormal HRT onset and slope, alongside factors such as smoking and hypertension (HT) (Table 5, Table 6, Table 7 and Table 8). Although the results suggest that smoking and HT contribute to abnormal HRT, no significant differences were observed between subgroups regarding these factors. The data indicate that individuals recovering from severe COVID-19 are at an increased risk of experiencing persistently blunted HRT, thereby elevating their likelihood of developing malignant ventricular arrhythmias compared to those who have never contracted COVID-19 or have only experienced mild to moderate cases. This enduring blunting of HRT may stem from the lasting direct impact of SARS-CoV-2, which affects the autonomic nervous system (ANS), the vagal nerve, myocardial tissue, and the intrinsic neural networks of the myocardium in the acute phase of the illness, or it could be related to the direct effects of pharmacological and invasive treatments (such as mechanical ventilation) employed for severe COVID-19 patients. HRV, HRT, is an important ECG-Holter-derived test that can be used to evaluate ANS dysfunction. Several recent studies have used HRV tests and their impairments to suggest that severe acute respiratory syndrome coronavirus 2 (SARS-CoV-2) infection may lead to ANS dysfunction [35,36]. A recent study examining the effects of the period after COVID-19 on HRT reported blunted HRT values in the group with a positive COVID-19 test history compared to the control group. This study, which was conducted by excluding patients with recovery from severe COVID-19, is not compatible with the results of patients who contracted and recovered from mild and moderate COVID-19 in our study. In our opinion, this may be related to the methodological way, exclusion criteria, and the selected sample size. This study compared the patient population with positive PCR results for active COVID-19 with the PCR-negative population [37]. In the current study, since we did not compare the post-recovery sample with those who had a positive PCR result for COVID-19, in other words, with active COVID-19 disease, with those who were PCR negative, the HRT results of cases recovering from mild and moderate COVID-19 were similar to those of PCR-negative cases. On the other hand, study results suggest that individuals in subgroup 3 have a higher probability of having blunted HRT and thus a higher predisposition to suffer from serious ventricular arrhythmias than the population outside this group. The main factors that distinguish the recovery from the severe COVID-19 subgroup from other subgroups in terms of HRT data are that the CNS and vagus nerve are affected more due to the increased viral load and the severity and duration of inflammation during active disease, the use of agents known to have harmful effects on the immune system and myocardium, such as steroids, in high doses (e.g., Prednisolone 1 g/day) and for a long time, and intubation of some severe cases due to prolonged deep hypoxia.

## 5. Conclusions

There has been a notable rise in the number of recovered COVID-19 individuals seeking help at psychiatric outpatient clinics due to complaints of palpitations. Recent studies attribute these palpitation complaints to conditions such as panic disorder, anxiety, or depression, and they include treatment algorithms in standard practice [38,39,40,41,42]. However, the persistence of palpitations, even months after recovery, raises concerns about the potential for lasting damage to the intrinsic conduction pathways, myocardium, or the autonomic nervous system (ANS). Any such damage could lead to arrhythmias and subsequently cause palpitations. The present study demonstrated a significant association between blunted heart rate turbulence (HRT) and recovery from severe COVID-19. In this context, the findings provide compelling evidence for the continued experience of palpitations months after COVID-19 treatment and highlight the importance of conducting thorough 24-h ECG-Holter monitoring. This proactive approach may facilitate the early detection of blunted HRT and help prevent malignant ventricular arrhythmias.

## Figures and Tables

**Table 1:** Semi-quantitative chest computed tomography (CT) severity score and chest CT severity index.

Individual Lobar Scores Based on Percentage of Involvement		COVID-19 Chest CT Severity Index Based on the Involvements of the FIVE lobes	
Lobar involvement	Score	Total Score	Severity (Category)
0–5%	1	<8	Mild
5–25%	2	8–15	Moderate
26–49%	3	16–25	Severe
50–75%	4		
>75%	5		

**Table 2:** Comparison of demographical and HRT parameters between the recovered COVID-19 group and control group.

	Control Group (Noun: 315)	Recovered COVID-19 Group (Noun: 253)	*p*
Age (years)	45.0 (26.0–55.0)	45.0 (33.0–52.0)	0.43
Gender (female) (%)	51.4	49.0	0.61
HT (%)	25.4	24.8	0.92
Smoking (%)	30.8	35.6	0.24
HRT onset (%)	−2.0 (−2.3–−1.4)	−1.5 (−2.4–−0.5)	<0.0001
HRT slope (ms/beat)	12.0 (9.0–15.0)	10.0 (8.0–15.0)	0.002
Abnormal HRT onset (%)	7.3	11.1	0.14
Abnormal HRT slope (%)	6.7	10.7	0.09
Category 0 (%)	89.8	83.4	0.02
Category 1 (%)	6.3	10.3	0.09
Category 2 (%)	3.8	6.3	0.18

**Table 3:** Comparison of HRT parameters between recovered COVID-19 subgroups and the control group.

	The Controls (Noun: 315)	Subgroup I (Recovered Mild COVID-19 Subgroup, Noun: 155)	Subgroup II (Recovered Moderate COVID-19 Subgroup, Noun: 56)	Subgroup III (Recovered Severe COVID-19 Subgroup, Noun: 42)	*p*
Age (years)	45.0 (26.0–55.0)	44.0 (32.0–50.0)	46.5 (35.25–55.75)	46.5 (40.0–54.25)	^a^ 1.0, ^b^ 0.71, ^c^ 0.26, ^d^ 0.65, ^e^ 0.22, ^f^ 0.99
Gender (female) (%)	51.4	50.3	48.2	45.2	^a^ 0.84, ^b^ 0.66, ^c^ 0.51, ^d^ 0.87, ^e^ 0.60, ^f^ 0.84
HT (%)	25.4	23.2	25.0	31.0	^a^ 0.65, ^b^ 1.0, ^c^ 0.45, ^d^ 0.85, ^e^ 0.32, ^f^ 0.65
Smoking (%)	30.8	34.8	35.7	38.1	^a^ 0.40, ^b^ 0.53, ^c^ 0.38, ^d^ 1.0, ^e^ 0.72, ^f^ 0.83
HRT onset (%)	−2.0 (−2.3–−1.4)	−2.0 (−2.6–−0.8)	−1.75 (−2.2–−1.0)	−1.0 (−1.6–−0.0)	^a^ 0.26, ^b^ 0.12, **^c^ <0.001**, ^d^ 0.95, **^e^ 0.01, ^f^ 0.04**
HRT slope (ms/beat)	12.0 (9.0–15.0)	10.0 (9.0–15.0)	10.75 (8.0–15.1)	8.5 (2.65–13.0)	^a^ 0.50, ^b^ 0.97, **^c^ <0.001**, ^d^ 1.0, **^e^ 0.01, ^f^ 0.04**
Abnormal HRT onset (%)	7.3	7.1	8.9	28.6	^a^ 1.0, ^b^ 0.88, **^c^ <0.001**, ^d^ 0.88, **^e^ <0.001**, ^f^ **0.02**
Abnormal HRT slope (%)	6.7	6.5	10.7	26.2	^a^ 1.0, ^b^ 0.42, **^c^ <0.001,** ^d^ 0.46, **^e^ <0.001**, ^f^ 0.06
Category 0 (%)	89.8	89.0	83.9	61.9	^a^ 0.87, ^b^ 0.24, **^c^ < =0.001,** ^d^ 0.34, **^e^ <0.001**, **^f^ 0.02**
Category 1 (%)	6.3	7.7	8.9	21.4	^a^ 0.56, ^b^ 0.67, ^c^ **0.002,** ^d^ 1.0,^e^ **0.02,** ^f^ 0.09
Category 2 (%)	3.8	3.9	5.4	16.7	^a^ 1.0, ^b^ 0.86, ^c^ **0.002**, ^d^ 0.93, ^e^ **0.01,** ^f^ 0.13

^a^: *p*-value between control group and subgroup 1; ^b^: *p*-value between control group and subgroup 2; ^c^: *p*-value between control group and subgroup 3; ^d^: *p*-value between subgroup 1 and subgroup 2; ^e^: *p*-value between subgroup 1 and subgroup 3; ^f^: *p*-value between subgroup 2 and subgroup 3.

**Table 4:** Spearman’s rho and Pearson’s correlation analyses between HRT onset, HRT slope, and some other variables.

Spearman’s Rho Correlation Analysis Between HRT Onset, HRT Slope Recovered COVID-19 Subgroups, Post COVID-19 Recovery Duration, Number of Positive PCR Tests for COVID-19				
	HRT Onset		HRT Slope	
	r	*p*	r	*p*
Recovered COVID-19 subgroups	0.191	0.002	−0.139	0.027
Pearson’s correlation analysis between HRT onset, HRT slope, and recovered COVID-19 subject’s chest CT severity score				
	HRT Onset		HRT Slope	
	r	*p*	r	*p*
Recovered COVID-19 subject’s chest CT severity score	0.251 < 0.001		−0.205	0.001
Post-COVID-19 recovery duration (week)	−0.051	0.421	−0.076	0.229
Number of positive PCR tests for COVID-19	0.010	0.874	−0.109	0.084

**Table 5:** Model 1: Binary logistic regression for variables (dependent variable: abnormal HRT onset).

	*β*	S.E.	Hazard Ratio	95% Confidence Interval		*p*
HT	1.335	0.456	3.802	1.556	9.289	0.003
Smoking	1.029	0.453	2.799	1.151	6.807	0.023
Age	0.005	0.020	1.005	0.967	1.045	0.800
Recovered severe COVID-19 subgroup	1.693	0.482	5.435	2.113	13.977	<0.001
Gender	−0.677	0.468	0.508	0.203	1.271	0.148
Post-COVID-19 recovery duration (week)	−0.003	0.005	0.997	0.987	1.006	0.479
Number of positive PCR tests for COVID-19	−0.463	0.496	0.630	0.238	1.664	0.351
Constant	−3.044	2.017	0.048			0.131
Model *p* < 0.001; Nagelke R^2^ = 0.252						
Recovered severe COVID-19: A person who contracted COVID-19, had a severe case, and then recovered						

**Table 6:** Model 2: Binary logistic regression for variables (dependent variable: abnormal HRT slope).

	*β*	S.E.	Hazard Ratio	95% Confidence Interval		*p*
HT	1.234	0.462	3.436	1.388	8.507	0.008
Smoking	0.683	0.457	1.979	0.809	4.843	0.135
Age	0.019	0.20	1.019	0.980	1.060	0.345
Recovered Severe COVID-19 Subgroup	1.559	0.487	4.756	1.830	12.356	0.001
Gender	−0.392	0.461	0.676	0.274	1.668	0.395
Post-COVID-19 recovery duration (week)	−0.009	0.005	0.991	0.982	1.000	0.060
Number of positive PCR tests for COVID-19	−0.661	0.460	0.516	0.209	1.273	0.151
Constant	−2.560	2.000	0.077			0.201
Model *p* < 0.001; Nagelke R^2^ = 0.234						

**Table 7:** Model 3: Linear regression for variables (dependent variable: HRT onset).

	Unstandardized Coefficients		Standardized Coefficients	t	*p*
	B	Std. Error	Beta		
Constant	−0.343	0.616		−5.557	0.578
HT	−0.409	0.160	−0.158	−2.2559	0.01
Smoking	−0.348	0.145	−0.149	−2.395	0.02
Age	0.002	0.006	0.023	0.366	0.71
Recovered COVID-19 subject’s chest CT severity score	0.035	0.009	0.240	3.916	<0.001
Gender	0.018	0.38	0.008	0.131	0.89
Post-COVID-19 recovery duration (week)	−0.003	0.002	−0.115	−1.873	0.06
Number of positive PCR tests for COVID-19	0.000	0.163	0.000	0.003	0.90
Model *p* < 0.001; Nagelke R^2^ = 0.345					

**Table 8:** Model 4: Linear regression for variables (dependent variable: HRT slope).

	Unstandardized Coefficients		Standardized Coefficients	t	*p*
	B	Std. Error	Beta		
Constant	7.441	2.894		2.571	0.01
HT	1.559	0.751	0.130	2.076	0.04
Smoking	1.556	0.682	0.143	2.282	0.02
Age	−0.017	0.026	−0.041	−0.658	0.51
Recovered COVID-19 subject’s chest CT severity score	−0.122	0.042	−0.179	−2.900	0.004
Gender	0.862	0.650	0.083	1.327	0.18
Post-COVID-19 recovery duration (week)	−0.004	0.008	−0.030	−0.479	0.63
Number of positive PCR tests for COVID-19	−1.260	0.767	−0.101	−1.644	0.10
Model *p* < 0.001; Nagelke R^2^ = 0.318					

## Data Availability

The data sets utilized and/or analyzed in this study can be obtained from the corresponding author upon a reasonable request.

## Abbreviations

ACE2	Angiotensin-converting enzyme 2
ANS	Autonomic nervous system
BMI	Body mass index
CNS	Central nervous system
COVID-19	Coronavirus 2 disease emerged in 2019
CT	Computed tomography
ECG	Electrocardiography
GIS	Gastrointestinal tract
HRT	Heart rate turbulence
HT	Hypertension
IL	Interleukin
LDH	Lactate dehydrogenase
PVC	Premature ventricular complex
SARS-CoV-1	Severe acute respiratory syndrome coronavirus 1
SARS-CoV-2	Severe acute respiratory syndrome coronavirus 2
TO	Turbulence onset
TS	Turbulence slope
